# Supplementary material for: A mathematical model of the impact of insulin secretion dynamics on selective hepatic insulin resistance
Source: Nat Commun. 2017 Nov 8;8:1362. doi: 10.1038/s41467-017-01627-9 (PMC5678123; doi:10.1038/s41467-017-01627-9)
Supplement: Supplementary file 1 — Supplementary Information [file 41467_2017_1627_MOESM1_ESM.pdf]

Supplementary information

Supplementary Methods

Model overview

The hepatic insulin signaling model included five signaling molecules, insulin receptor (R), IRS1 (S_1), IRS2 (S_2), Akt (B), and aPKC (C). The model was fitted to two published data sets simultaneously (described in the main text). The pre-hepatic insulin level has not been measured in both data sets. The estimation of pre-hepatic insulin, namely the input to the model, is also described below. All the simulation work was done based on SBPD toolbox for Matlab [1]. The bifurcation analysis was done with Matcont [2]. Matlab code for generating main figures in the main text and main Supplementary Figures are included in Supplementary Software 1.

Given the fact that the number of unknown parameters is more than that of experimental data points, we took a parsimonious manner in designing the model in order to have minimal number of unknown parameters. Firstly, for those processes that no data is available, multiple intermediary processes were lumped into one kinetic term. This applied to insulin receptor and IRS1/2 activation. Secondly, for feedbacks that have overlapping effects, we implemented only one that is upstream to others. This applied to Akt, since Akt has been reported to positively feedback to insulin receptor [3], IRS1 [4] and itself [5]. In this case, only the feedback from Akt to insulin receptor was implemented in the model (Akt auto-feedback was also tested). Lastly, interactions, including feedback and crosstalk mechanisms, are represented in the model by Hill functions of the following forms:

$$\mathrm{H_a}(p,x) = p + (1-p)\frac{x^2}{1+x^2}$$

$$\mathrm{H_i}(p,x) = 1 - (1-p)\frac{x^2}{1+x^2}$$

$$x = \frac{X}{m}$$

where the subscripts a and i indicate activation and inhibition, respectively. p is in the range [0, 1]. X represents the concentration of the molecule that carries out the action, m is the half-maximal concentration.

Model

Insulin receptor (R)

The insulin receptor is activated following insulin (I) binding. With physiological levels of insulin (<5nM), binding of a second insulin molecule on the receptor is rare and therefore is neglected here. The ligand binding and the following auto-phosphorylation of the receptor are lumped into one kinetic term ($r_{1\mathrm{f}}$). Dephosphorylation of activated receptor ($r_{1\mathrm{b}}$) is regulated by Akt (B_a) mediated feedback. The activated receptor (R_p) then undergoes internalization ($r_{2\mathrm{f}}$), which is regulated by aPKC (C_a). Internalized receptor undergoes dephosphorylation ($r_{3\mathrm{f}}$), which is also regulated by Akt (B_a), and further reinsertion ($r_{4\mathrm{f}}$). At physiological levels of insulin, most of the internalized receptors recycle to the plasma membrane [6]. Therefore, the degradation of internalized receptors was not considered. The kinetic terms and the equations concerning insulin receptors are:

$$R + I \rightleftarrows R_{\mathrm{p}} \quad r_1 = r_{1\mathrm{f}} R I - r_{1\mathrm{b}} \, \mathrm{H_i}(r_{1\mathrm{p}}, \frac{B_{\mathrm{a}}}{r_{1\mathrm{m}}}) R_{\mathrm{p}}$$

$$R_{\mathrm{p}} \rightarrow R_{\mathrm{p,en}} \quad r_2 = r_{2\mathrm{f}} R_{\mathrm{p}} \, \mathrm{H_a}(r_{2\mathrm{p}}, \frac{C_{\mathrm{a}}}{r_{2\mathrm{m}}})$$

$$R_{\mathrm{p,en}} \rightarrow R_{\mathrm{en}} \quad r_3 = r_{3\mathrm{f}} \, \mathrm{H_i}(r_{1\mathrm{p}}, \frac{B_{\mathrm{a}}}{r_{1\mathrm{m}}}) R_{\mathrm{p,en}}$$

$$R_{\mathrm{en}} \rightarrow R \quad r_4 = r_{4\mathrm{f}} R_{\mathrm{en}}$$

$$\frac{dR}{dt} = -r_1 + r_4$$

$$\frac{dR_{\mathrm{p}}}{dt} = -r_2 + r_1$$

$$\frac{dR_{\mathrm{p,en}}}{dt} = -r_3 + r_2$$

$$\frac{dR_{\mathrm{en}}}{dt} = -r_4 + r_3$$

IRS1 (S_1)

There is evidence that the total amount of IRS1 protein is not changed by postprandial insulin stimulation [7]. This allowed us to model only activation and deactivation processes of IRS1, and to use the fraction of activated IRS1 ($S_{1\mathrm{a}}$) as a variable in the model.

IRS1 is activated ($b_{0\mathrm{f}}$) and deactivated ($b_{0\mathrm{b}}$) via tyrosine phosphorylation and dephosphorylation by the insulin receptor and phosphatases, respectively. The activity of IRS1 is also influenced by multiple downstream effectors in the insulin signaling network, such as Akt (B_{a}) and aPKC (C_{a}), which can phosphorylate multiple serine/threonine residuals on IRS1 [8]. Importantly, serine/threonine phosphorylation on IRS1 can either improve or impair signaling. As the positive feedback from Akt on the receptor has already been implemented, only negative effect from Akt is included here. For aPKC, we set up two parallel models where the effect of aPKC on IRS1 is positive and negative,

respectively. Numerical studies showed that only a negative effect from aPKC on IRS1 could fit the data sets. The kinetic terms and equations for IRS1 read:

$$
\begin{aligned}
&S_1 \rightleftarrows S_{1a} \quad r_{b0} = b_{0f}(R_p + R_{p,en})\,\mathrm{H_i}(b_{0,p1}, \frac{C_a}{b_{0,m1}})\,\mathrm{H_i}(b_{0,p2}, \frac{B_a}{b_{0,m2}})(1 - S_{1a}) - b_{0b}S_{1a} \\
&\frac{dS_{1a}}{dt} = r_{b0}
\end{aligned}
$$

IRS2 (S_2)

Unlike IRS1, the total amount of IRS2 protein showed remarkable decrease after feeding due to transcriptional inhibition by Akt-FoxO [7]. Consequently, the synthesis (c_{0f}) of IRS2, which is regulated by Akt (B_a), is included in the model. The activation of IRS2 (c_{1f}) is modeled by one kinetic term. Activated IRS2 (S_{2a}) undergoes degradation (c_{2f}). The kinetic terms and equations for IRS2 read

$$
\begin{aligned}
&null \rightarrow S_2 \qquad r_{c0} = c_{0f}\,\mathrm{H_i}(c_{0p}, \frac{B_a}{c_{0m}}) \\
&S_2 \rightarrow S_{2a} \qquad r_{c1} = c_{1f}(R_p + R_{p,en})S_2 \\
&S_{2a} \rightarrow null \quad r_{c2} = c_{2f}S_{2a} \\
&\frac{dS_2}{dt} = r_{c0} - r_{c1} \\
&\frac{dS_{2a}}{dt} = r_{c1} - r_{c2}
\end{aligned}
$$

aPKC (C)

There is evidence that the amount of aPKC in rodent liver is unchanged after feeding (Farese R. V. private communication). Therefore only the (de-)activation processes (d_{0f} and d_{2f}) are modeled and the fraction of activated aPKC (C_a) is used as a variable. aPKC is activated only by the IRS2 branch [9]. The activation process requires IRS2/P13K

binding, (auto-)trans-phosphorylation and a conformational change (d_{1f}) [10,11]. The state before the conformational change is denoted by C_t. The kinetic terms and equations for aPKC read:

$$
\begin{aligned}
&C \rightleftarrows C_{\mathrm{t}} && r_{\mathrm{d0}} = d_{\mathrm{0f}}\,\mathrm{H}_{\mathrm{a}}(d_{\mathrm{0,p}}, \frac{C_{\mathrm{a}}}{d_{\mathrm{0,m}}}) S_{2,\mathrm{a}} (1 - C_{\mathrm{t}} - C_{\mathrm{a}}) - d_{\mathrm{0b}} C_{\mathrm{t}} \\
&C_{\mathrm{t}} \rightarrow C_{\mathrm{a}} && r_{\mathrm{d1}} = d_{\mathrm{1f}} C_{\mathrm{t}} \\
&C_{\mathrm{a}} \rightarrow C && r_{\mathrm{d2}} = d_{\mathrm{2f}} C_{\mathrm{a}} \\
&\frac{dC_{\mathrm{t}}}{dt} = r_{\mathrm{d0}} - r_{\mathrm{d1}} \\
&\frac{dC_{\mathrm{a}}}{dt} = r_{\mathrm{d1}} - r_{\mathrm{d2}}
\end{aligned}
$$

Akt (B)

Akt is activated by both the IRS1 and IRS2 branches and is inhibited by aPKC. There is evidence that the total amount of Akt is kept constant during postprandial insulin stimulation [7]. As in the case of IRS1, the activated fraction of Akt (B_a) is used as variable and only the activation (e_{0f}) and deactivation (e_{0b}) terms are present in the Akt equation. The kinetic terms and the equations for Akt read:

$$
\begin{aligned}
&B \rightleftarrows B_{\mathrm{a}} \quad r_{\mathrm{e0}} = e_{\mathrm{0f}}\,\mathrm{H}_{\mathrm{i}}(e_{\mathrm{0,p0}}, \frac{C_{\mathrm{a}}}{e_{\mathrm{0,m0}}})\,\mathrm{H}_{\mathrm{a,a}}(e_{\mathrm{0,p1}}, \frac{S_{1,\mathrm{a}}}{e_{\mathrm{0,m1}}}, \frac{S_{2,\mathrm{a}}}{e_{\mathrm{0,m2}}})(1 - B_{\mathrm{a}}) - e_{\mathrm{0b}} B_{\mathrm{a}} \\
&\frac{dB_{\mathrm{a}}}{dt} = r_{\mathrm{e0}}
\end{aligned}
$$

Estimation of rodent postprandial pre-hepatic insulin level

Information on rodent pre-hepatic vein insulin levels is rare. We found two publications in which both plasma and pre-hepatic insulin level have been measured simultaneously [12,13]. The data are summarized in the following Table.

site / condition	plasma (pM)	pre-hepatic base/peak (pM)	fraction of pulsatile insulin
12 hour fast	256	200/630	60%
0.5 hour after hyperglycemic clamp	800	900/4500	60%

We assumed parabola relationships between plasma insulin level and the peak/base level of pre-hepatic pulsatile insulin. With those values from literature and the extrapolation limit point (0,0), we obtained the following relationships

$$B_{\text{hep,ins}} = 0.000993 I_{\text{plas}}^2 + 0.591 I_{\text{plas}}$$
$$A_{\text{hep,ins}} = 0.003068 I_{\text{plas}}^2 + 2.995 I_{\text{plas}}$$
$$I_{\text{hep,pul}} = B_{\text{hep,ins}} + A_{\text{hep,ins}} \left| \sin\left(\frac{\pi}{P} t\right) \right|$$
$$I_{\text{hep,npul}} = B_{\text{hep,ins}} + \frac{2}{\pi} A_{\text{hep,ins}}$$

where I_{plas} denotes plasma insulin level, $B_{\text{hep,ins}}$ denotes basal level of pre-hepatic pulsatile insulin, $A_{\text{hep,ins}}$ denotes the amplitude of pre-hepatic pulsatile insulin, $I_{\text{hep,pul}}$ denotes pre-hepatic pulsatile insulin, P denotes the period of the pulses, $I_{\text{hep,npul}}$ denotes pre-hepatic non-pulsatile insulin, which was used in the simulation of the feeding experiment. With above formulas, the fraction of pulsatile pre-hepatic insulin is in the range 60%-70%, which is consistent with reported values in the literature. Plasma insulin level was taken from [7] to build the insulin input for the refeeding experiment while

insulin levels for the infusion experiments was assumed to be the insulin level 1 hour after the beginning of feeding in the refeeding experiment.

Simulation

We fitted the model to the two data sets simultaneously. Specifically, in numerical optimization, we tried to minimize the root mean square (RMS) difference between model simulation (y) and the measured data points (ŷ),

$$RMS = \sqrt{\sum_{j=1}^{n_{\mathrm{r}}} \frac{(y_{\mathrm{r,j}} - \hat{y}_{\mathrm{r,j}})^2}{\sigma_{\mathrm{r,j}}^2} + \sum_{i=1}^{n_{\mathrm{i}}} \frac{(y_{\mathrm{i,j}} - \hat{y}_{\mathrm{i,j}})^2}{\sigma_{\mathrm{i,j}}^2}}$$

where suffix r and i denote refeeding and infusion experiments respectively, σ denotes the standard deviation of the measured data point, and *n* denotes the number of data points.

All the simulation work was done based on the SBPD toolbox for Matlab. The SBPD toolbox was modified in parts for our special purpose. A differential evolution based global optimizer was employed to fit the parameters in the model. We allowed a large parameter space for the fitting procedure to search. Each optimization task was repeated 50 times. The optimizer returns a population of parameters that give rise to acceptable fitting results. We took not only the best fitting result for further analyses, but also those fitting results that are acceptable based on the Akaike information criterion with correction (AICc), with a relative likelihood of more than 0.37 (corresponding to 2 units difference in AICc). The definition of AICc reads

$$AICc = 2P + RMS^2 + N\log(2\pi) + 2\frac{P(P+1)}{N-P-1}$$

where P is the number of fitted parameters, N is the number of data points.

We further made sure that the fitting results for analysis were well separated in terms of Euclidean distance, by filtering out inferior solutions (in terms of RMS) residing in the vicinity, defined by the relative distance filter (rDF) d_r<0.1, of a superior one. In particular, the relative distance d_r between two parameter vectors is defined as

$$d_r = \sqrt{\frac{1}{P}\sum_{k=1}^{P}\frac{(v_{i,k}-v_{j,k})^2}{v_{j,k}^2}}$$

where suffix i and j are index for parameter vectors with $\mathbf{V}_i$ being inferior in terms of RMS, k is the index for dimension within parameter vector, P is the number of dimension of parameter vectors (i.e. number of fitted parameters).

We also investigated the response of the fitted model to variations in insulin levels and quantified the robustness to variations in insulin levels by the mean of AICc (mAICc), which is computed based on model responses to different insulin doses. The responses of the fitted model to 31 different insulin levels, linearly sampled in the range from 85% to 115%, were used to calculate mAICc. Models, as well as fitting results, were finally ranked by mAICc. A relative likelihood of more than 0.37 (corresponding to 2 units difference in mAICc) is used in gating.

Parameter identifiability analysis

We performed parameter identifiability analysis based on the minimal model. Considering that measurements of IRS1/2 and Akt were present in the data sets used for fitting while no measurement of insulin receptor or aPKC is available, we carried out the

identifiability analysis for parameters directly involved in insulin receptor and aPKC. In particular, the parameter boundary [0.01 100], which was used in the previous fittings for determining the minimal model, was divided to four bins: [0.01 0.1], [0.1 1], [1 10] and [10 100] in order to generate diverse solutions. The model was fitted with one particular parameter being confined to one of the four bins while boundaries for other parameters were as before. Each fitting was repeated five times. This was done for eight parameters: r_{1f}, r_{1b}, r_{2f}, r_{3f}, r_{4f}, d_{0f}, d_{0b} and d_{2f}. For d_{1f} which represents the rate of the conformational change of the partially activated aPKC molecule, the boundary [50 500] was divided into 2 bins: [50 100] and [100 500] and corresponding optimization tasks were performed. Altogether, the identifiability analysis consists of 170 optimization tasks (8x4x5+2x5), the results of which were further analyzed in the following steps.

At first, we checked insulin receptor dynamics. Previous *in vitro* experimental studies using metabolic active rat hepatocytes indicated that insulin receptor internalization and recycling can quickly follow physiological levels of insulin pulses [14]. However, we have found that some of the fitted results were associated with slow receptor internalization and recycling such that insulin receptors cannot follow 5-min pulses of insulin (see an example in Supplementary Figure 7). These fitting results were excluded from further analysis.

Secondly, we applied the AICc criteria, which resulted in 4396 acceptable parameter sets. Thirdly, we applied the relative distance filter, which reduce the number of parameter sets to 528. And finally, we applied the mAICc criteria, which resulted in 13 parameter sets. The distribution of each parameter after each step was presented in Supplementary Figure 2 (b) and (c).

In principle, the genetic algorithm we used converges to the global minimum when running time approaches infinity [15]. However in practice, the algorithm has to be terminated by empirical rules. Consequently, the global minimum was likely not identified in our numerical studies.

The identifiability analysis showed that 14 parameters were well confined in relative small ranges (both the ratio between the maximum and the median, and the ratio between the median and the minimum were smaller than two, see Supplementary Figure 1). The other 11 parameters were not well confined (at least one of the ratios was larger than two, see Supplementary Figure 1).

Fitted Parameter values

Fitted Parameter values are provided in Supplementary Data 1, including the 13 parameter sets, as gated by mAICc, for M4 in the identifiability analysis.

Matlab codes for generating main figures

Matlab codes for generating main figures in both the main text and the Supplementary Information are provided in Supplementary Software 1.

Supplementary Figures

Supplementary Figure 1. Distribution of fitted parameters in the minimal model

Supplementary Figure 1 (a)

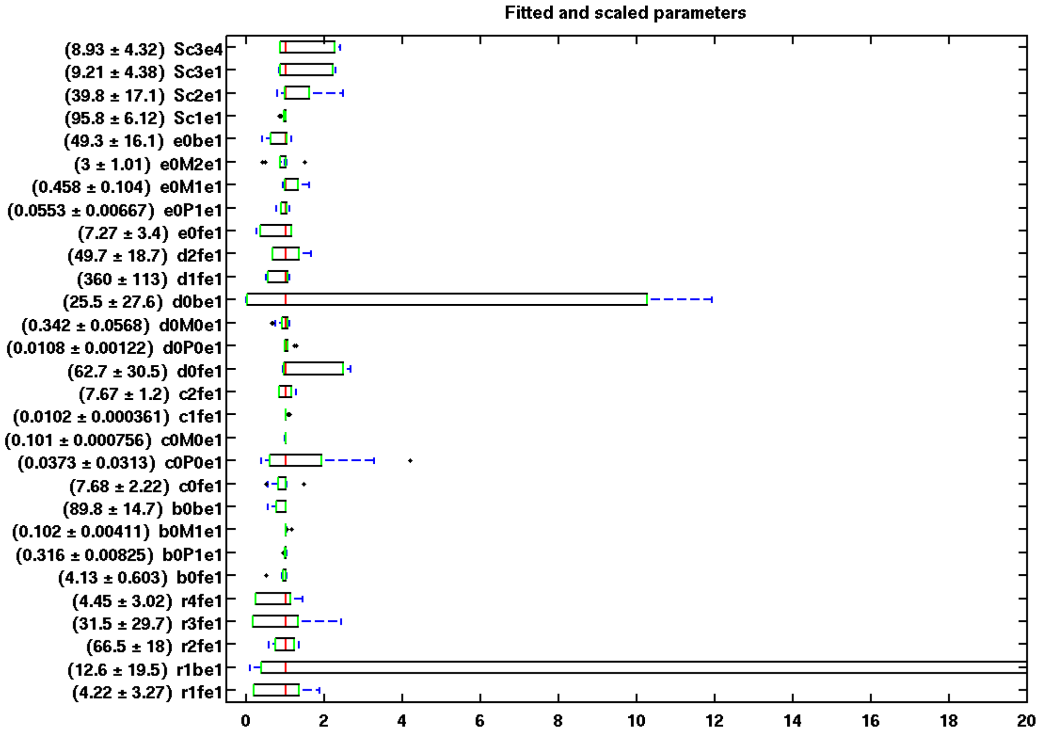

Supplementary Figure 1 (b)

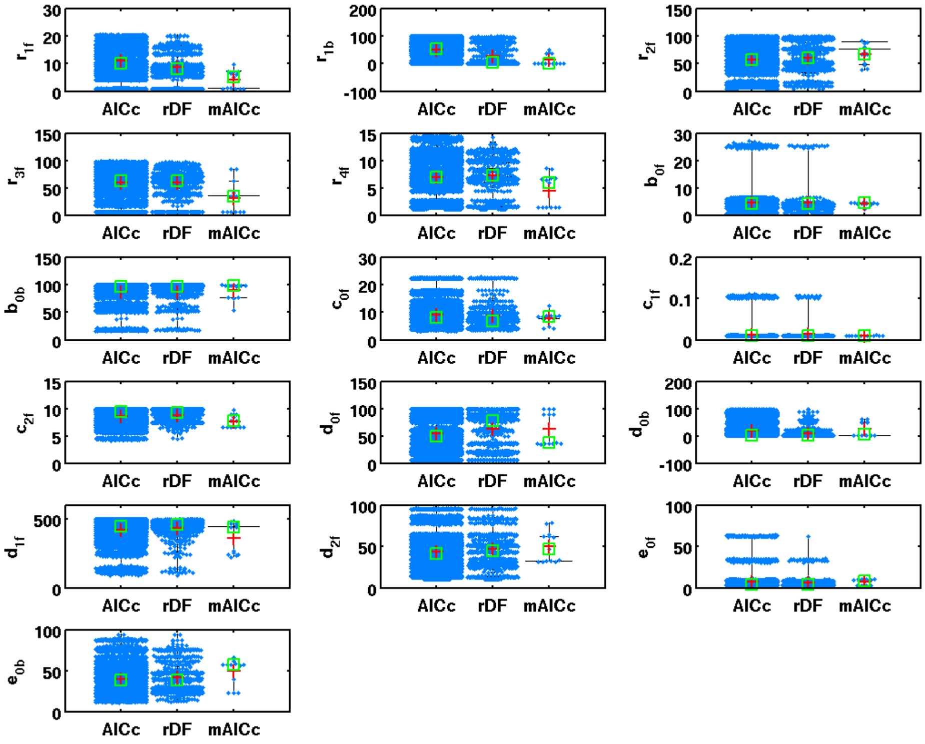

Supplementary Figure 1 (c)

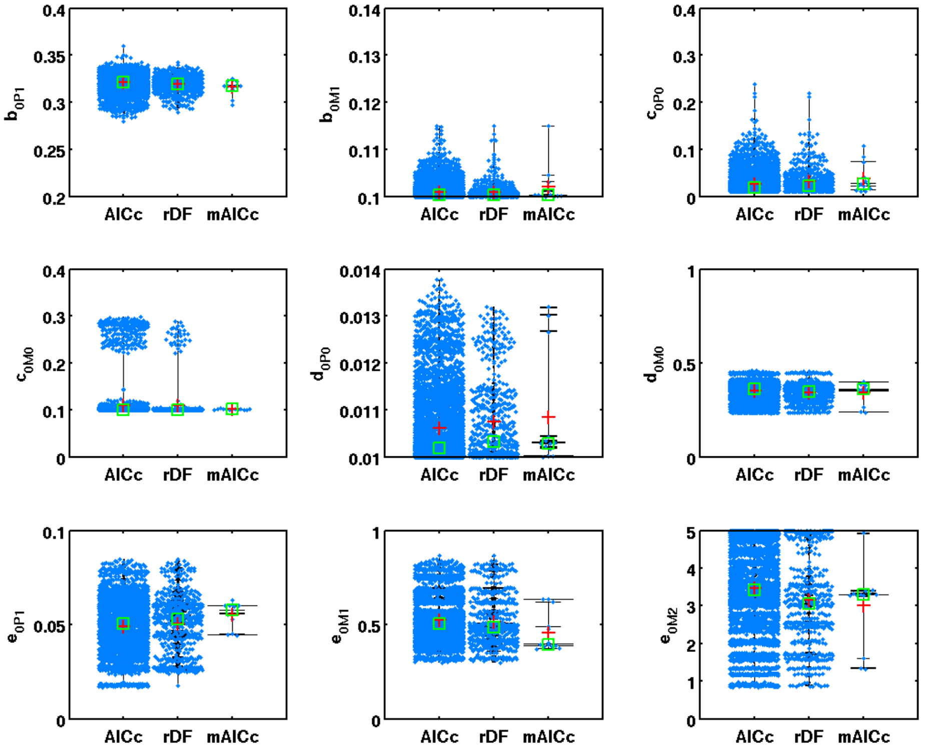

Supplementary Figure 1 (a) Box and whisker plot for fitted parameters from the identifiability analysis. Each parameter is normalized such that the median value is 1 (red). The upper and lower quartiles (p_{75} and p_{25}) are marked by green. The range, from p_{25}-1.5(p_{75}-p_{25}) to p_{75}+1.5(p_{75}-p_{25}), is marked by blue. Mean ± standard deviation for each parameter are shown along the labels of the vertical axis. The x-axis is truncated at 20 to show more detail in the small range. The maximum of parameter r_{1b} reaches 400. (b) and (c), distribution of parameters resulting from AICc, relative distance filter (rDF), and mAICc criterion in the identifiability analysis. Mean and median values are marked by red cross and green square respectively. (b) shows kinetic parameters, (c) shows parameters in various Hill functions.

Supplementary Figure 2. Multiple fitting results (related to Fig. 2 in the main text)

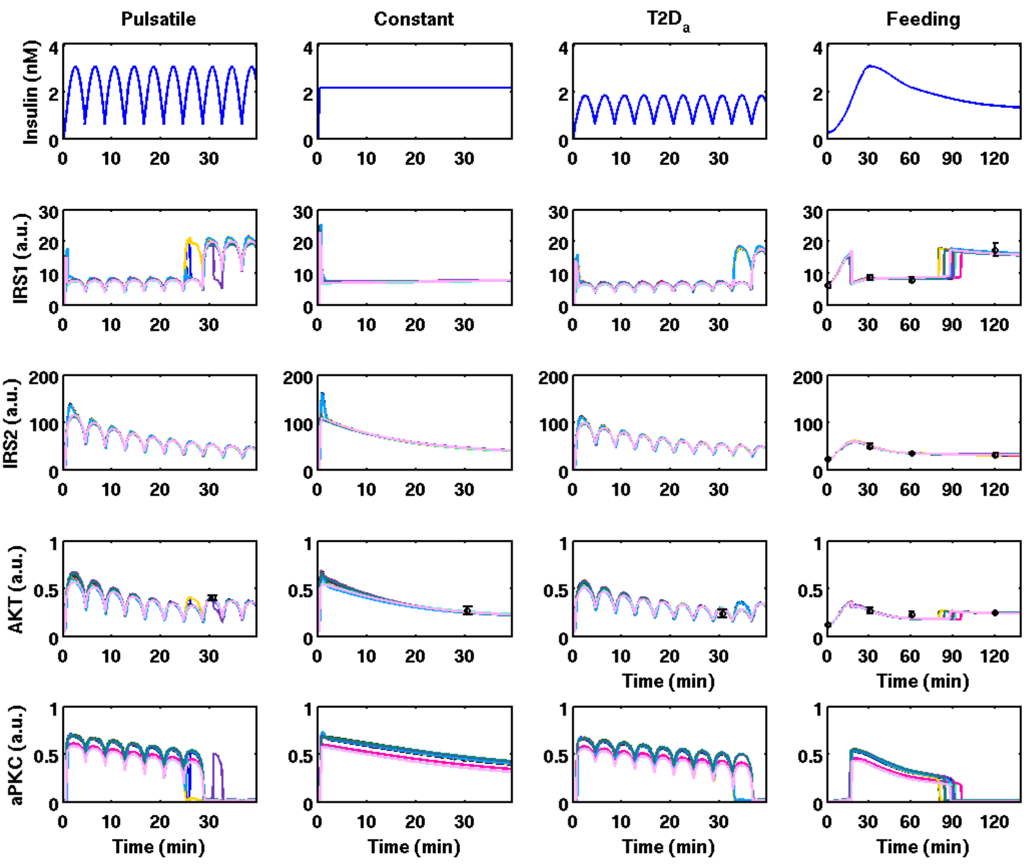

Supplementary Figure 2 Multiple fitting results, coded by color, of the minimal model. The associated 13 parameter sets are included in the xls file in the supplement. Experimental data are indicated by black circles with error bars (s.e.m). The main difference is the timing of the aPKC switch.

Supplementary Figure 3 Multiple fitting results of M3

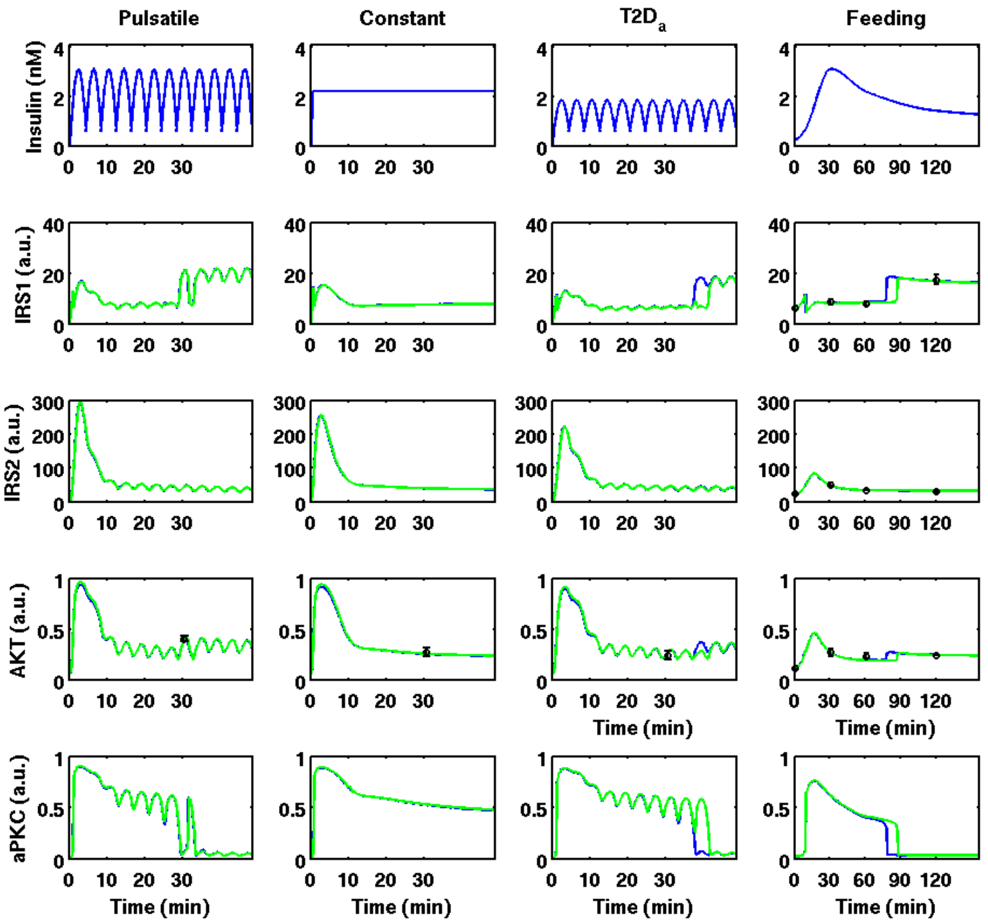

Supplementary Figure 3, Two fitting result (green and blue), gated by mAICc, of M3.

Supplementary Figure 4. Model response to different insulin doses is robust (related to Fig. 4 in the main text)

Supplementary Figure 4 (a)

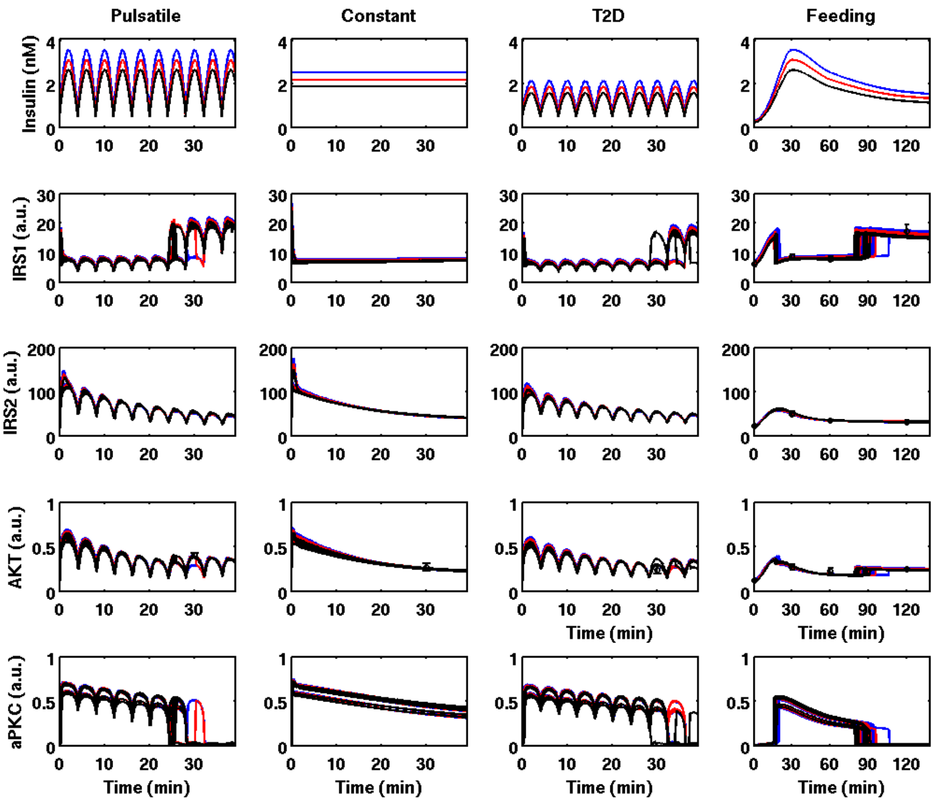

Supplementary Figure 4 (b)

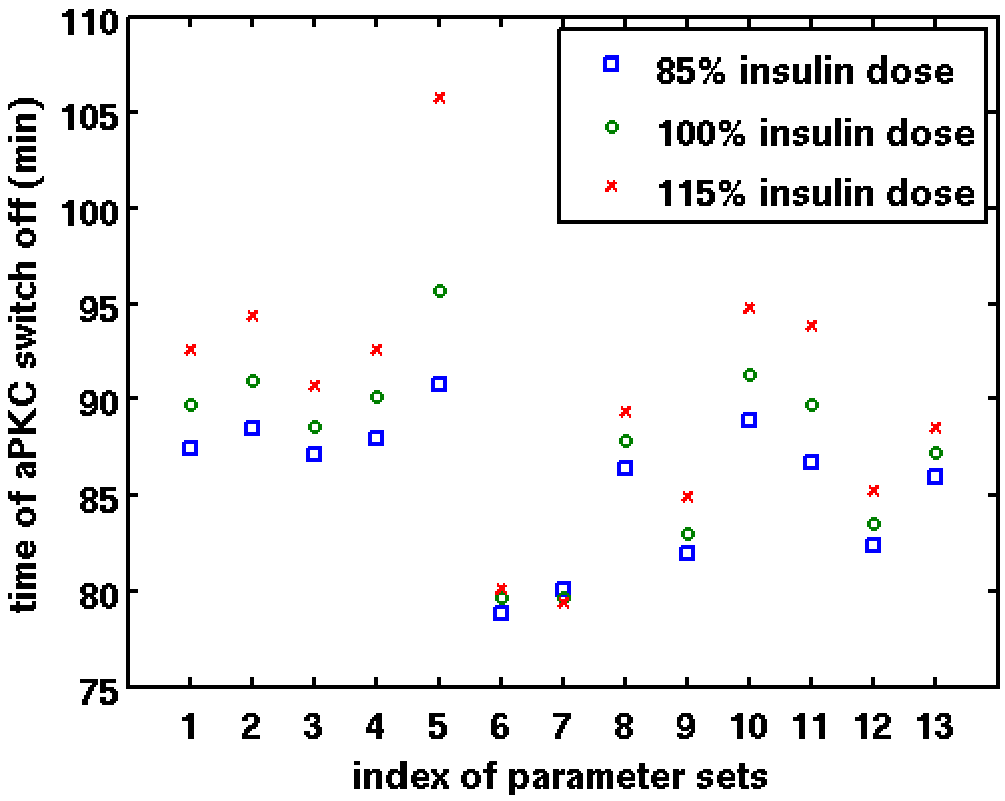

Supplementary Figure 4 (c)

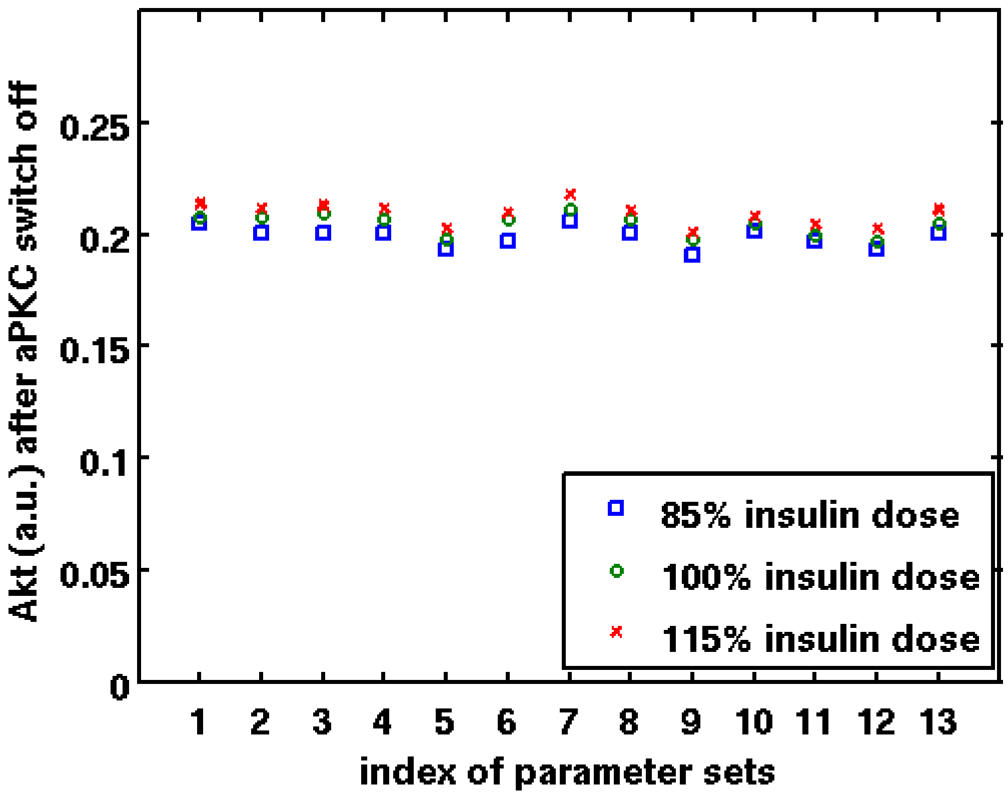

Supplementary Figure 4. Response of the minimal model, based on the 13 parameter sets resulting from the identifiability analysis, to different insulin doses are shown (a). The time of aPKC switch-off (b) and the level of Akt rebound (c) are also shown.

Supplementary Figure 5. Response to insulin profiles with different dynamic features (related to Fig. 5 in the main text)

Supplementary Figure 5 (a)

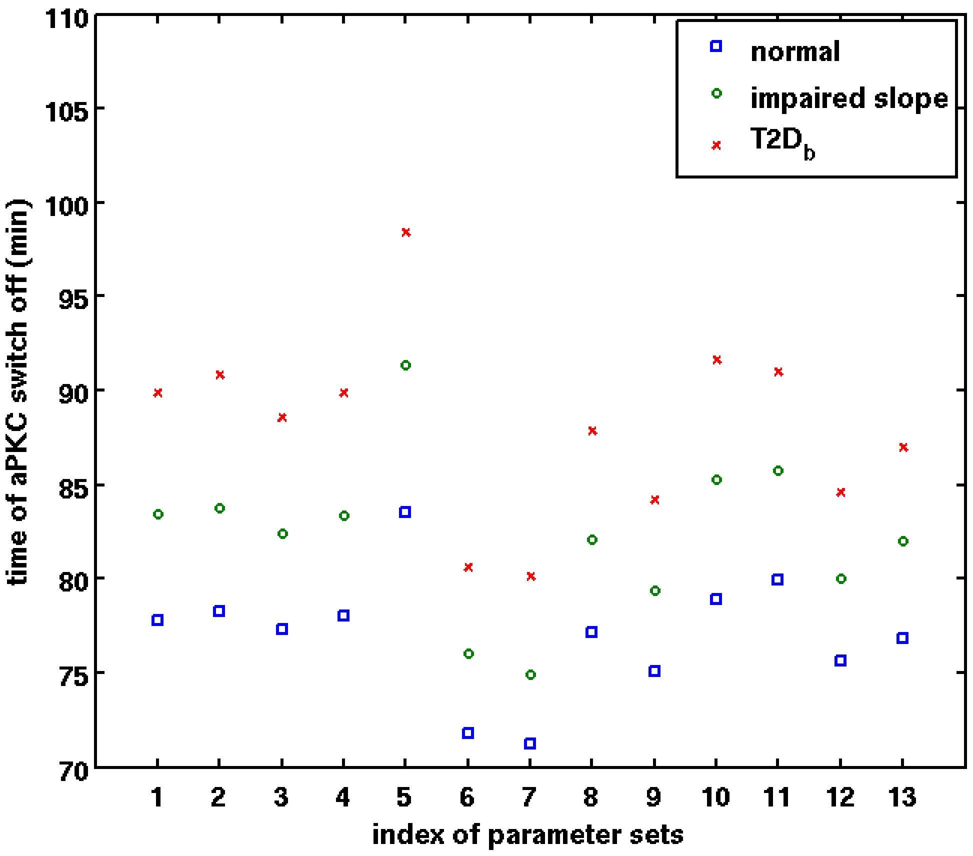

Supplementary Figure 5 (b)

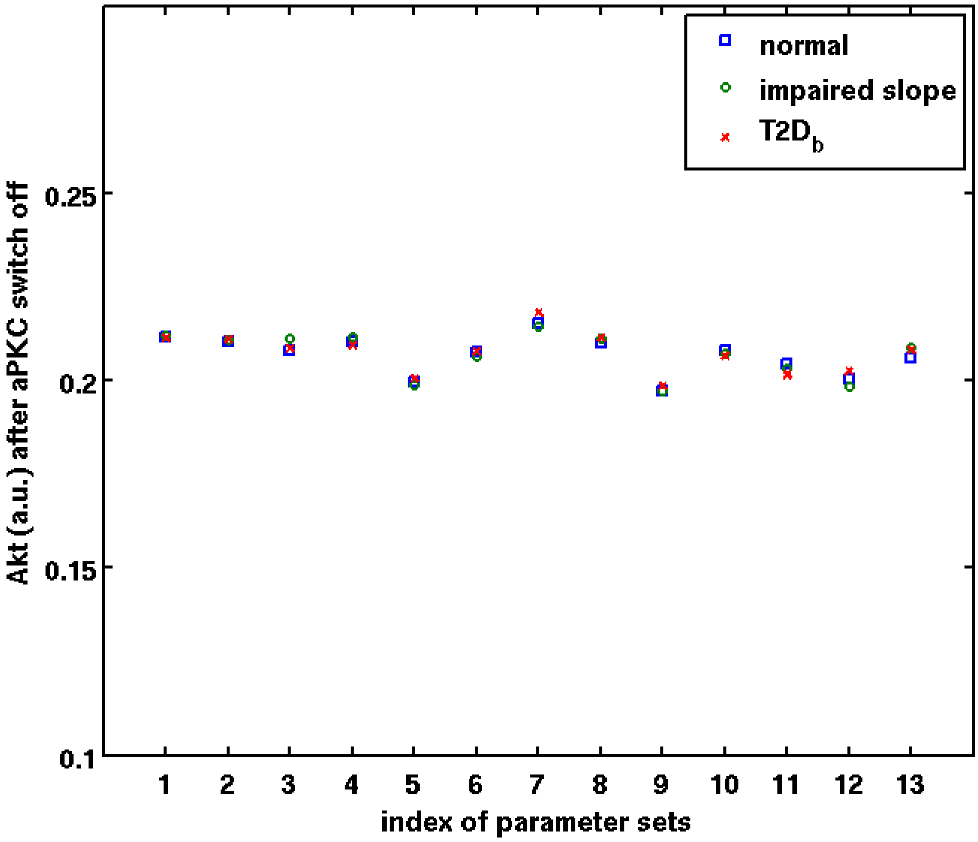

Supplementary Figure 5 (c)

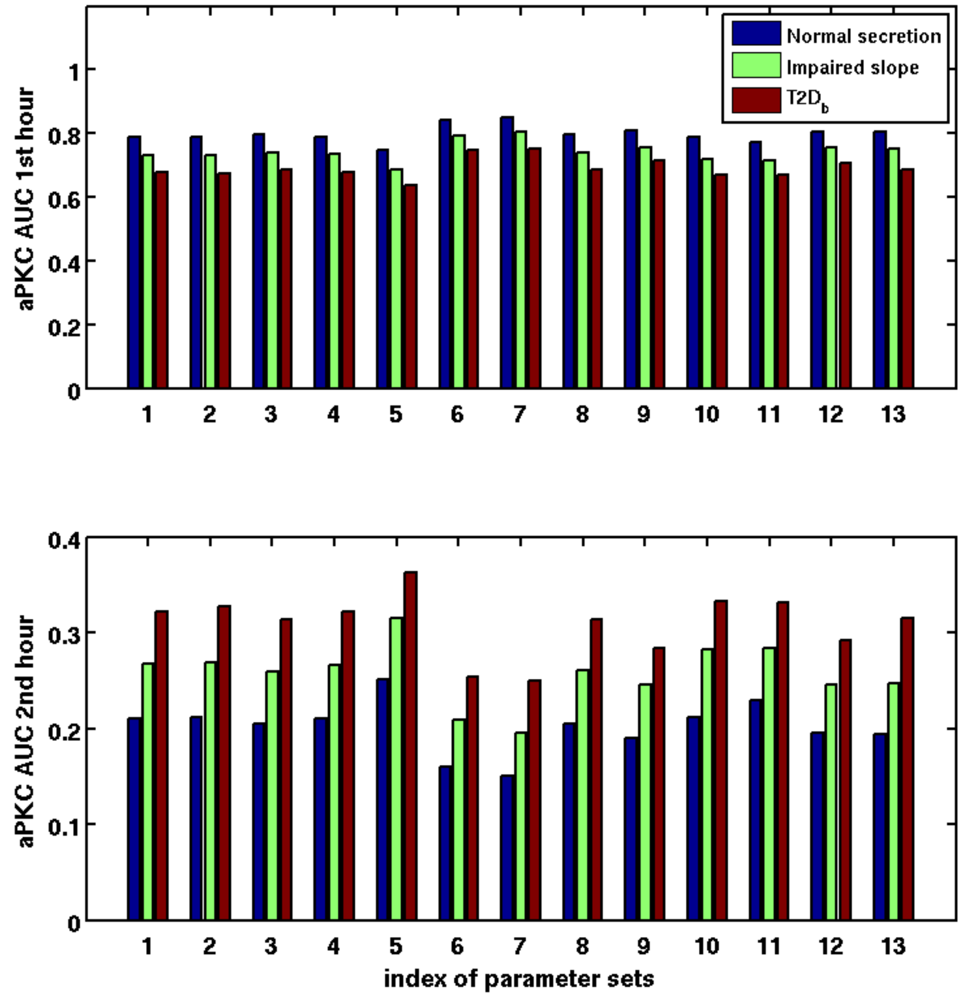

Supplementary Figure 5, The response of the minimal model to insulin profiles with different dynamical features. The time of aPKC switch-off (a), the level of Akt rebound (b) and the AUC of aPKC in first and second hour (c) are shown.

Supplementary Figure 6. Response to different pulsatile insulin (related to Fig. 6 in the main text)

Supplementary Figure 6 (a)

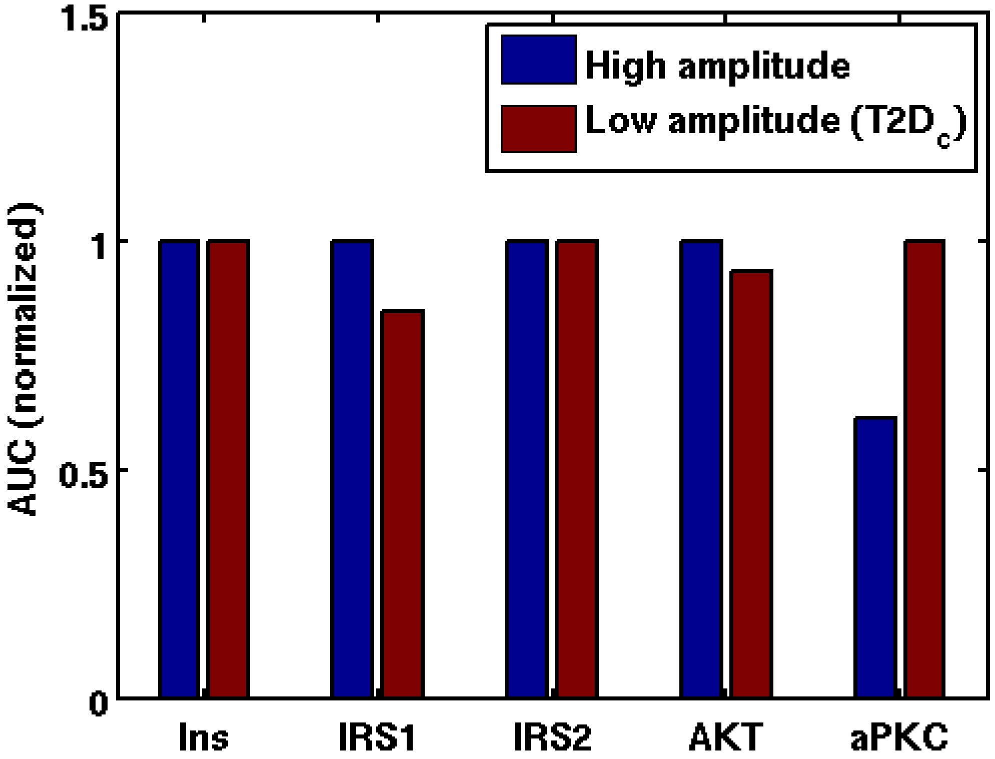

Supplementary Figure 6 (b)

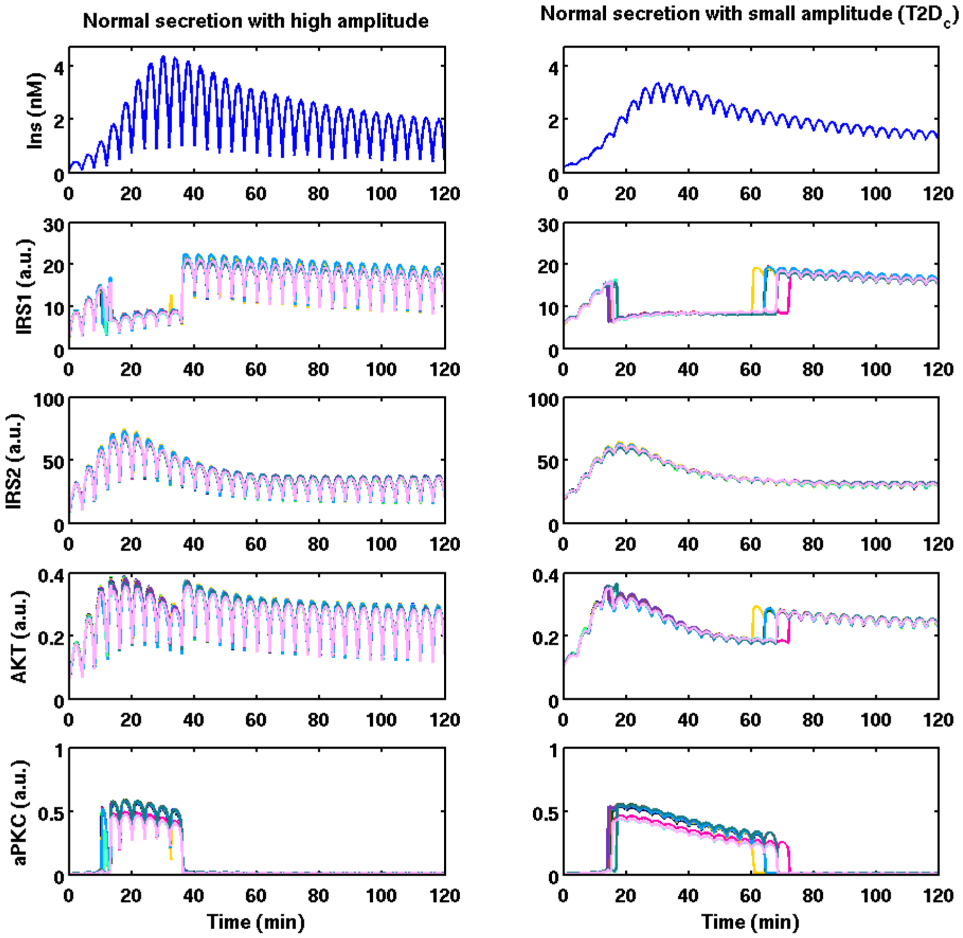

Supplementary Figure 6 (a), Area under curve, corresponding to Fig. 6 in the main text, is compared for high and low amplitude of insulin pulses. The trend here is similar to Fig.5F in the main text. (b) Response of the model to pulsatile insulin with high and low amplitude. Multiple simulation results, coded by color, are shown. The associated 13 parameter sets are included in the xls file in the supplement.

Supplementary Figure 7 Two patterns of insulin receptor dynamics

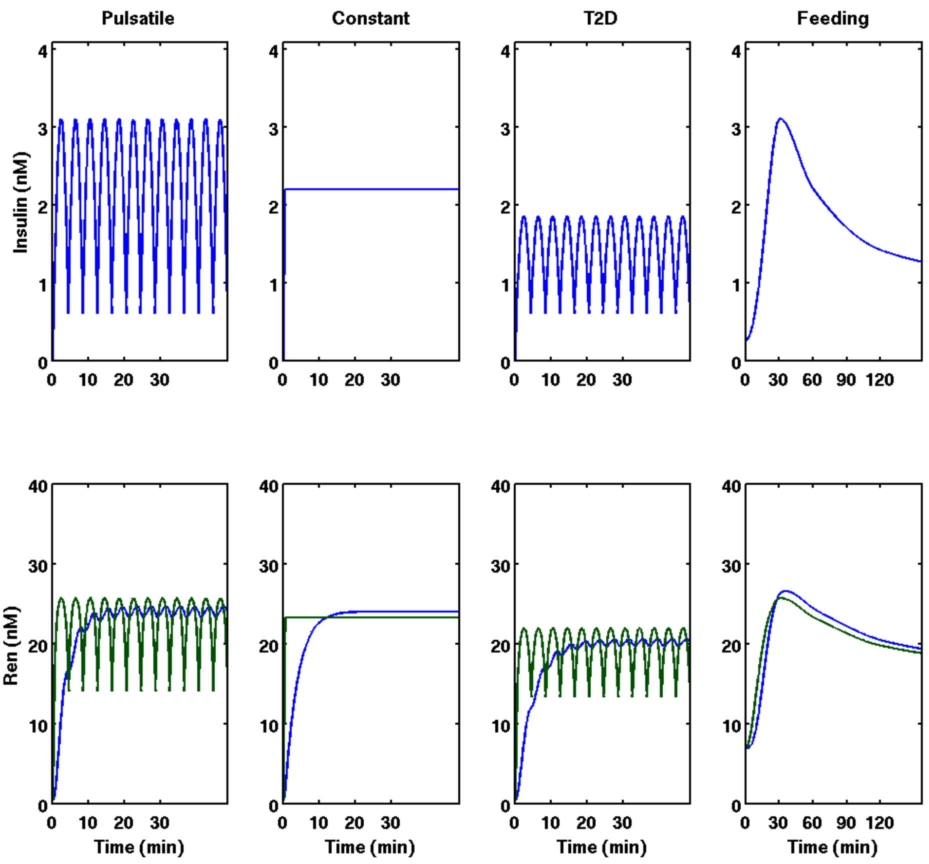

Supplementary Figure 7 Example of the two patterns of insulin receptor dynamics in the simulation of infusion experiments. Although the two curves of insulin receptor (bottom row) are similar in the refeeding experiment (rightmost column), they are different in the infusion experiments. Insulin receptor can either follow insulin pulses (green curves, consistent with published experiments) or not (blue curves, contrary to published experiments). Parameter sets associated with the fast pattern of insulin dynamics were further analyzed.

Supplementary References

1. Schmidt, H. SBaddon: high performance simulation for the Systems Biology Toolbox for MATLAB. *Bioinformatics* **23,** 646–647 (2007).
2. Dhooge, A., Govaerts, W. & Kuznetsov, Y. A. MATCONT: A MATLAB package for numerical bifurcation analysis of ODEs. *ACM Trans. Math. Softw.* **29,** 141–164 (2003).
3. Ravichandran, L. V., Chen, H., Li, Y. & Quon, M. J. Phosphorylation of PTP1B at Ser50 by Akt impairs its ability to dephosphorylate the insulin receptor. *Mol. Endocrinol.* **15,** 1768–1780 (2001).
4. Luo, M. *et al.* Phosphorylation of Human Insulin Receptor Substrate-1 at Serine 629 Plays a Positive Role in Insulin Signaling. *Endocrinology* **148,** 4895–4905 (2007).
5. Ozcan, L. *et al.* Activation of Calcium/Calmodulin-Dependent Protein Kinase II in Obesity Mediates Suppression of Hepatic Insulin Signaling. *Cell Metab.* **18,** 803–815 (2013).
6. Guglielmo, G. M. D., Baass, P. C., Authier, F., Posner, B. I. & Bergeron, J. J. M. Insulin receptor internalization and signalling. *Mol. Cell. Biochem.* **182,** 59–63 (1998).
7. Kubota, N. *et al.* Dynamic Functional Relay between Insulin Receptor Substrate 1 and 2 in Hepatic Insulin Signaling during Fasting and Feeding. *Cell Metab.* **8,** 49–64 (2008).
8. Taniguchi, C. M., Emanuelli, B. & Kahn, C. R. Critical nodes in signalling pathways: insights into insulin action. *Nat Rev Mol Cell Biol* **7,** 85–96 (2006).

9. Farese, R. V., Sajan, M. P. & Standaert, M. L. Insulin-Sensitive Protein Kinases (Atypical Protein Kinase C and Protein Kinase B/Akt): Actions and Defects in Obesity and Type II Diabetes. *Exp. Biol. Med.* **230,** 593–605 (2005).
10. Farese, R. V. & Sajan, M. P. Metabolic functions of atypical protein kinase C: “good” and “bad” as defined by nutritional status. *Am. J. Physiol. - Endocrinol. Metab.* **298,** E385–E394 (2010).
11. Standaert, M. L., Bandyopadhyay, G., Kanoh, Y., Sajan, M. P. & Farese, R. V. Insulin and PIP3 Activate PKC-ζ by Mechanisms That Are Both Dependent and Independent of Phosphorylation of Activation Loop (T410) and Autophosphorylation (T560) Sites†. *Biochemistry (Mosc.)* **40,** 249–255 (2001).
12. Matveyenko, A. V., Veldhuis, J. D. & Butler, P. C. Measurement of pulsatile insulin secretion in the rat: direct sampling from the hepatic portal vein. *Am. J. Physiol. - Endocrinol. Metab.* **295,** E569–E574 (2008).
13. Matveyenko, A. V., Veldhuis, J. D. & Butler, P. C. Adaptations in pulsatile insulin secretion, hepatic insulin clearance, and β-cell mass to age-related insulin resistance in rats. *Am. J. Physiol. - Endocrinol. Metab.* **295,** E832–E841 (2008).
14. Goodner, C. J., Sweet, I. R. & Harrison, H. C. Rapid Reduction and Return of Surface Insulin Receptors After Exposure to Brief Pulses of Insulin in Perifused Rat Hepatocytes. *Diabetes* **37,** 1316–1323 (1988).
15. Ghosh, S., Das, S., Vasilakos, A. V. & Suresh, K. On Convergence of Differential Evolution Over a Class of Continuous Functions With Unique Global Optimum. *IEEE Trans. Syst. Man Cybern. Part B Cybern.* **42,** 107–124 (2012).
