# Supplementary material for: A mathematical model of the impact of insulin secretion dynamics on selective hepatic insulin resistance
Source: Nat Commun. 2017 Nov 8;8:1362. doi: 10.1038/s41467-017-01627-9 (PMC5678123; doi:10.1038/s41467-017-01627-9)
Supplement: Supplementary file 3 — Description of Additional Supplementary Files [file 41467_2017_1627_MOESM3_ESM.pdf]

Description of Supplementary Files

File name: Supplementary Data 1
Description: Multiple parameter values of the minimal model.

File name: Supplementary Software
Description: Matlab codes for generating main figures.
